# Cold Atmospheric Plasma Exerts Antimicrobial Effects in a 3D Skin Model of Cutaneous Candidiasis

**DOI:** 10.3390/antibiotics12050933

**Published:** 2023-05-19

**Authors:** Sarah Fink, Michael Fischer, Sebastian Spange, Oliver Beier, Kerstin Horn, Jörg Tittelbach, Cornelia Wiegand

**Affiliations:** 1 Department of Dermatology, Jena University Hospital, Friedrich Schiller University, 07747 Jena, Germany; 2 Institute of Micro- and Nanotechnologies, Ilmenau University of Technology, 98639 Ilmenau, Germany; 3 Innovent e.V. Jena, 07745 Jena, Germany

**Keywords:** 3D skin model, cold atmospheric plasma, *C. albicans*

## Abstract

Cutaneous candidiasis is characterized by an overgrowth of *Candida* leading to skin inflammation and infection. Similar to bacteria, *Candida* can develop tolerance to common antifungal drugs. Cold atmospheric plasma (CAP), with its proven antimicrobial properties, offers a promising alternative to the prevailing methods. Because of plasma heterogeneity each new device must be tested individually for its effectiveness. Antimicrobial activity is usually studied using planktonic microorganisms or animal models, making it difficult to extrapolate the results to the human system. Therefore, a 3D skin model of cutaneous candidiasis for the antimicrobial testing of CAP was established. First, the reaction of the 3D-skin model to *Candida* infection was examined using various histological and molecular–biological methods. Infection with *C. albicans* resulted in increased expression and secretion of pro-inflammatory cytokines and augmented expression of antimicrobial peptides. Within 48 h, hyphal growth spread throughout the model and caused tissue damage. Second, the CAP treatment was employed. It was shown that CAP significantly reduced the spread of the yeast in the infected skin models as well as decreased the expression and secretion of the infection markers. The plasma device exhibited a high antifungal activity by completely inhibiting hyphal growth and reducing inflammation at the highest treatment duration.

## 1. Introduction

Cutaneous candidiasis is a common disease mainly caused by *C. albicans* in humans [1]. The illness is typically characterized by an itchy, patchy reddening associated with exudation and maceration. White scaled satellites can be found at the edges. Numerous small pustules appear as the primary efflorescence. The lesions can cause burning and pain, especially if the skin is eroded. In healthy people, spread is prevented by the skin microbiome [2]. In immunosuppression, diabetes, or long-term treatment with antibiotics, *C. albicans* can overgrow the skin [1]. Azole antifungals are often preferred for topical treatment of *Candida* infections. However, the intrinsic or acquired resistance of *C. albicans* to azoles is often observed [3]. This requires the establishment of new alternative therapies.

In the recent years, cold atmospheric plasma has become an alternative tool for antimicrobial treatment. Plasma is known as the fourth state of matter and is a partially ionized gas. It consists of ions, electrons, uncharged particles, and radicals. There are two types of plasma: thermal (hot) and non-thermal (cold) plasma. In thermal plasma, uncharged molecules and electrons are at an energy equilibrium and have the same very high temperature. Examples of hot plasma are the corona of the sun, plasma in a fusion reactor, or a discharge arc such as in welding or arc lamps. In medicine, thermal plasmas are mainly used as surgical tools [4]. In cold plasma, only electrons exhibit a high energy level and because of the mass difference between the electrons and the uncharged molecules, the resulting plasma has a low temperature of about 40 °C [4,5]. These temperatures allow the safe and painless treatment of human tissues [6,7].

Because of its excellent antimicrobial effect, CAP represents an alternative to antibiotics for wound treatment [8]. However, the antimicrobial effect depends on the type of plasma generation (plasma jet, dielectric barrier discharge), the working gas, or the treatment distance and duration, as these factors affect the formation and concentration of reactive species in CAP that mediate the biological effects. CAP mainly consists of reactive species such as free radicals, reactive molecules (e.g., ozone and hydrogen), negative and positive ions, as well as electrons. High levels of reactive species can lead to oxidative stress in prokaryotic and eukaryotic cells, causing necrotic or apoptotic cell death. Moreover, depending on the device, varying amounts of UV radiation are produced. Therefore, the antimicrobial effect of each plasma source needs to be studied individually [5]. The plasma source investigated in this study is based on the LTCC (low-temperature cofired ceramic) technology [9] and generates a planar, homogeneous atmospheric plasma. As such, a CAP source can be customized to any shape and size, and it could be of great interest for clinical applications. *Candida* infections of the skin can lead to severe limitations in the general well-being of patients in a hospital but also in older patients with various comorbidities in nursing homes [1]. In addition, treatment of cutaneous candidiasis is made more difficult by resistance to antimycotics [3].

Initial assessments of antimicrobial testing are based on planktonic microorganisms or biofilm studies [10]. These test systems do not take into account the skin microenvironment or a pro-inflammatory response [11]. Animal models offer an alternative test system. However, the differences in skin structure as well as the immune system limit the transferability of the data [12]. In addition, the political and social pressure to develop alternatives to animal models according to the 3R principle (reduction, refinement, and replacement of animal models) is growing [13]. In vitro approaches such as 3D skin disease models have recently gained interest not only because of the increased awareness of marked interspecies differences that impede the effective translation of results from animal models to humans but also in light of the implementation of the 3R principle [13,14]. Hence, it was the aim of this study to establish a suitable 3D model for cutaneous candidiasis that can be employed to test the antimicrobial effect of CAP treatment against *C. albicans*. To the best of our knowledge, this is the first study to describe Candida infection and CAP treatment in 3D-human skin models.

## 2. Results

### 2.1. Plasma Generation Using LTCC Technology

**Figure 1:**
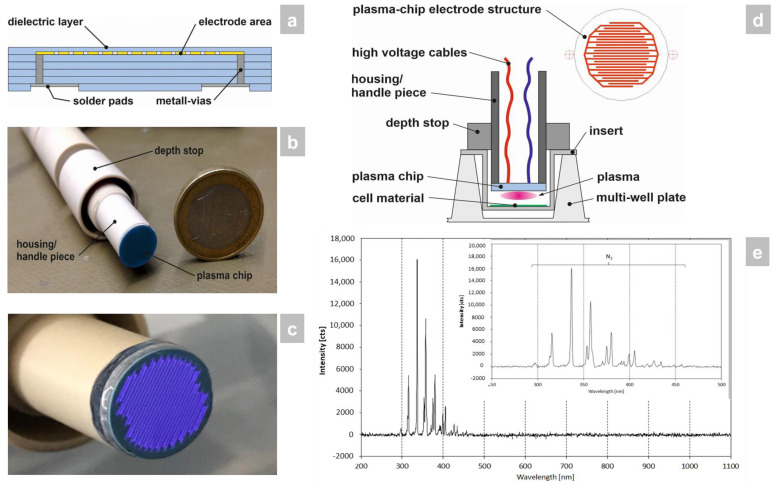
Manufacture and surveying of the CAP device used in the study. (**a**) The LTCC plasma chip was constructed by multilayer build-up from individual unsintered ceramic foils containing screen-printed microelectrodes, metallic vias, solder pads, and a dielectric barrier through lamination and sintering. (**b**) The LTCC plasma chip was then installed in a pen-like hand piece, which was sized to fit a 24-well plate format for the in vitro study that was carried out. (**c**) The CAP source generates a planar, homogeneous atmospheric plasma using ambient air. (**d**) The hand piece contains the power connector for the LTCC chip to generate the CAP and has a depth stop to set the exact distance to the 3D skin models with candidiasis. (**e**) Optical emission spectrum of the plasma source. The integration time collecting the emissions was 1 s and the distance between plasma source-OES sensor head was around 5 mm. The plasma discharge was ignited within ambient air and a power of approx 5 W.

Low-temperature cofired ceramic (LTCC) technology [9] was employed for the construction of the plasma chip because of its excellent dielectric material properties, high mechanical and chemical resistance, as well as good microstructurability. In this process, a so-called multilayer was built up from individual unsintered ceramic foils containing screen-printed microelectrodes, metallic vias, solder pads, and a dielectric barrier (Figure 1a). The multilayer assembly was subsequently laminated and sintered. The outer geometry of the ceramic plasma chip was created by laser structuring. In order to be able to carry out the effectiveness tests in the best possible way, the shape of the device was adapted to 24-well plates (including inserts) for this study (Figure 1b). The CAP source generated a planar, homogeneous atmospheric plasma (Figure 1c). The plasma chip was connected to the power source and built into a pen-like hand piece (Figure 1b,d).

Within the investigated wavelength range of 200–1100 nm only plasma emissions between 290 and 460 nm occurred (Figure 1e). All emission lines were assigned to the second positive system of N_2_. No additional oxygen-containing species were exited, such as NO, O, or OH, which could be expected for air as ambient gas (NO, O) or plasma interactions with air humidity (OH).

### 2.2. Determination of Microbial Growth and Cell Damage

**Figure 2:**
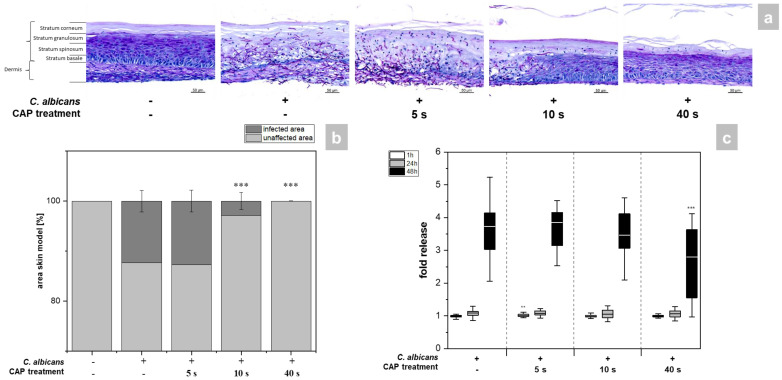
(**a**) Histological analysis of hyphal growth: 3D skin models were infected with *C. albicans* and treated with CAP for 5, 10 and 40 s. *C. albicans* hyphal growth was visualized after 48 h using PAS staining. (**b**) Graphic evaluation hyphal growth after 48 h: The infected area was determined as a percentage of the total area of the skin model using the PAS-stained histological sections. (**c**) Cytotoxicity analysis: After infection and CAP treatment of the skin models, the release of LDH in the supernatant was determined using the LDH assay. The fold release is calculated based on comparison with the untreated, non-infected control at the same point in time. Statistically significant differences were determined by comparison of the infected skin models without treatment and the CAP-treated models at the same point in time. Asterisks indicate significant deviations from the respective control: ** *p* ≤ 0.01 and *** *p* ≤ 0.001.

The skin models exhibited a fully differentiated epidermis consisting of stratum basale, stratum spinosum, stratum granulosum, and stratum corneum (Figure 2a). The thickness of the dermis was less pronounced compared to other full skin models [15] as no additional matrix was provided for the skin model [16,17]. PAS (periodic acid–Schiff) staining allowed easy distinction of the hyphal growth of *C. albicans* in the infected models. A total of 48 h after infection, strong growth of hyphae was evident, which was associated with distinct tissue damage (Figure 2a). CAP treatment resulted in a time-dependent reduction of *C. albicans* hyphal growth (Figure 1a). While short CAP treatment (5 s) did not affect hyphal growth, longer treatment periods (40 s) completely prevented hyphal propagation in the treated area and decreased tissue damage. *C. albicans* hyphae reduction was found to be significant (*p* < 0.001) after 10 and 40 s CAP treatment (Figure 2b). The results were confirmed by LDH measurement (Figure 2c). This enzyme is released in the supernatant of the skin models when the cell membrane is damaged and significantly reduced amounts were detected after 40 s CAP treatment at 48 h (*p* < 0.001, Figure 2c). (Figure S1).

### 2.3. Expression and Secretion of Pro-Inflammatory Cytokines

**Figure 3:**
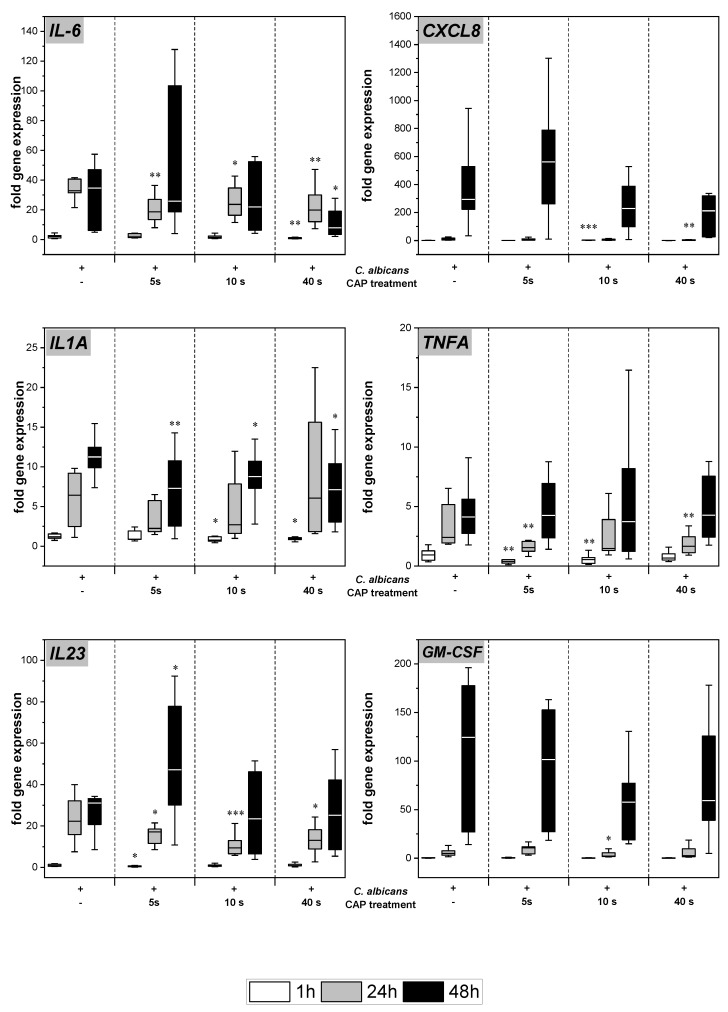
Expression of pro-inflammatory cytokines: The gene expression of the cytokines *IL6*, *CXCL8*, *IL1A*, *TNFA*, *IL23*, and *GM-CSF* was determined using RT-qPCR. The relative expression was calculated based on the untreated, non-infected control at the same point in time. Statistically significant differences were determined by comparison of the infected skin models without treatment and the CAP-treated models at the same point in time. Asterisks indicate significant deviations from the respective control: * *p*-values ≤ 0.05, ** *p* ≤ 0.01, and *** *p* ≤ 0.001.

*C. albicans* infection of the skin models resulted in increased gene expression of various pro-inflammatory cytokines (Figure 3). Subsequent to CAP treatment, significantly decreased levels of *IL-6* (*p* < 0.05), *TNFA* (*p* < 0.01), and *IL23* (*p* < 0.05) gene transcripts were noted after 24 h. At 48 h, the reduction was only observed at the highest CAP dose. For *IL-1A*, a reduction of gene expression levels was observed after 48 h for all CAP treatment durations (*p* < 0.05). In the cases of *IL8* and *GM-CSF*, a treatment duration-dependent decline in gene expression was noted at 48 h, but results did not reach statistical significance.

**Figure 4:**
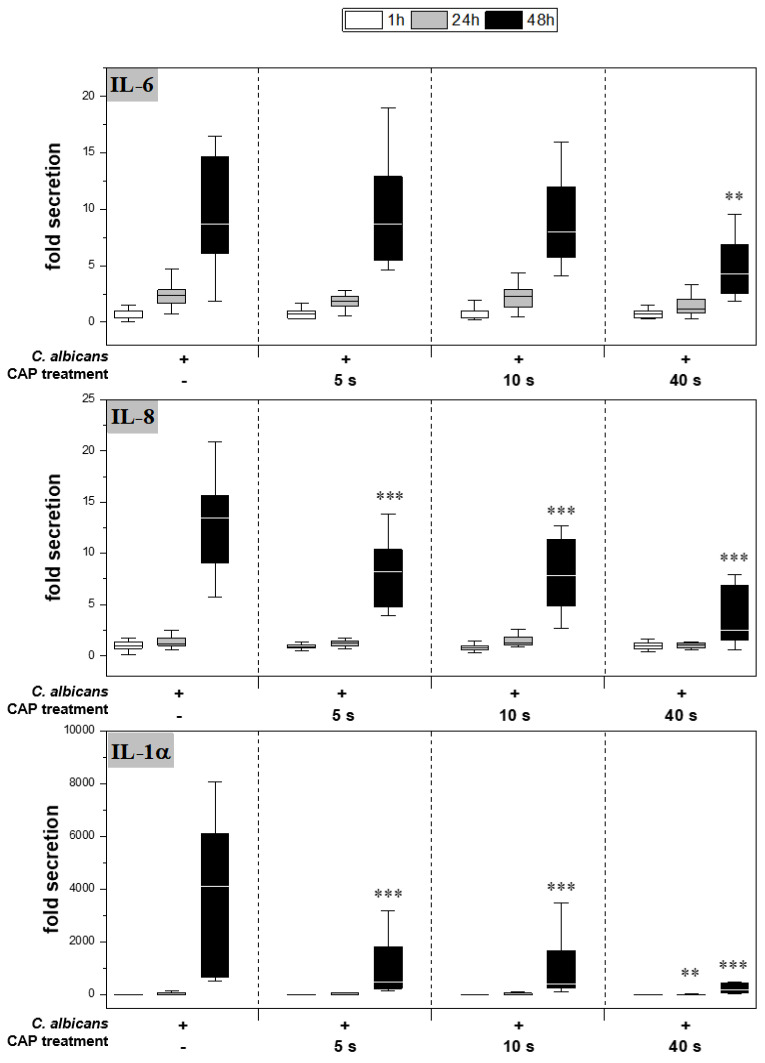
Secretion of pro-inflammatory cytokines: After infection of the skin models and treatment with plasma, the release of IL-6 (A), IL-8 (B) and IL-1α (C) was determined by ELISA. The fold secretion was calculated based on the untreated, uninfected control at the same point in time. Statistically significant differences were determined by comparison of the infected skin models without treatment and the CAP-treated models at the same point in time. Asterisks indicate significant deviations from the respective control: ** *p* ≤ 0.01 and *** *p* ≤ 0.001.

CAP further reduced cytokine secretion compared to the infected skin models without treatment (Figure 4). For IL-6, a significant reduction was observed only after 48 h of 40 s CAP treatment (*p* < 0.01). In contrast, a highly significant decrease in IL-8 (*p* < 0.001) and IL-1α (*p* < 0.001) release was noted at 48 h for all CAP treatments.

### 2.4. Expression of Antimicrobial Peptides

**Figure 5:**
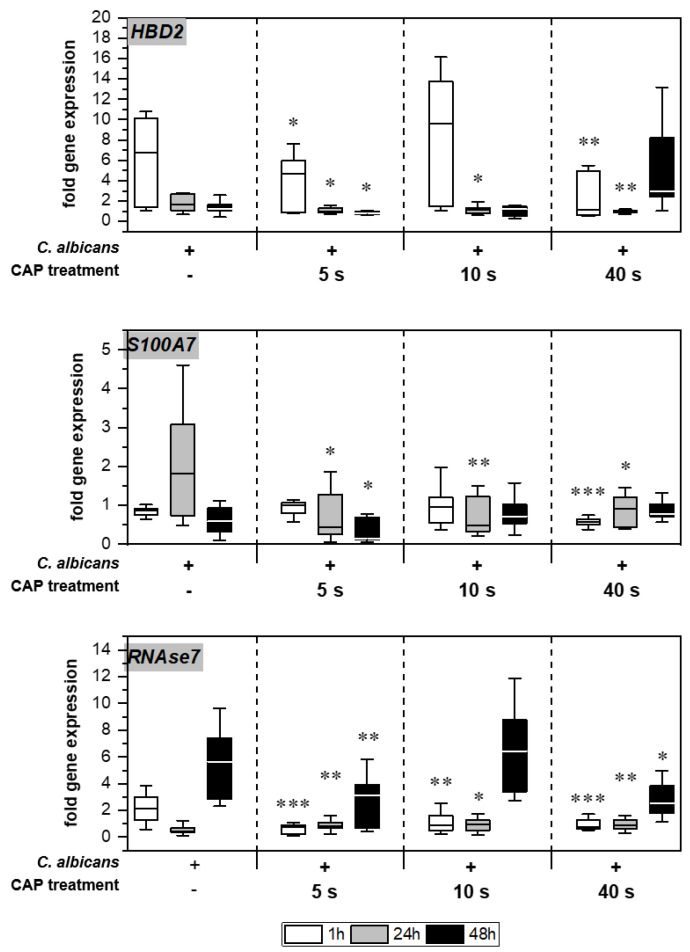
Expression of antimicrobial peptides: The gene expression of the AMPs *HBD2*, *S100A7*, and *RNAse7* was determined using RT-qPCR. The relative expression was calculated based on the untreated, non-infected control at the same point in time. Statistically significant differences were determined by comparison of the infected skin models without treatment and the CAP-treated models at the same point in time. Asterisks indicate significant deviations from the respective control: * *p*-values ≤ 0.05, ** *p* ≤ 0.01 and *** *p* ≤ 0.001.

Without treatment, Candida infection led to enhanced expression of different genes associated with host immune response in the skin models (Figure 5). Infection response was detected as early as 1 h after C. albicans infection, indicated by the distinctly increased HBD2 expression noticeable for the untreated infection control. CAP treatment of the infected skin models resulted in a significant decrease in HBD2 gene expression levels (*p* < 0.05). CAP treatment of the infected skin models resulted in a significant decrease in HBD2 gene expression levels up to 24 h (*p* < 0.05), with the exception of 10 s CAP treatment at 1 h. In addition, a subsequent increase in HBD2 levels after 40 s CAP treatment was noted. Moreover, significantly reduced gene expression was also observed for S100A7 (*p* < 0.05) and RNASE7 (*p* < 0.05) up to 48 h in some cases.

## 3. Discussion

Cold atmospheric plasma has emerged as an effective antimicrobial tool in recent years, particularly for wound treatment. However, the antimicrobial effect can vary owing to different types of devices, working gases, treatment times, etc. Therefore, the antimicrobial effectiveness must be determined individually. In this study a 3D skin model of cutaneous candidiasis was developed, which allowed the investigation of plasma efficiency in a complex microenvironment.

*C. albicans* is an opportunistic pathogen that generally occurs as a harmless commensal of the skin [18]. Nonetheless, this pathogen is a common cause of symptomatic skin infections and one of the main causes of life-threatening nosocomial infections [19]. Since the stratum corneum of the skin represents a barrier that is difficult to overcome, it can only be passed through active penetration. *C. albicans* can enter the skin through hyphal growth and activation of proteases. The invasiveness varies within different strains. The strain used in this study is an invasive growing clinical isolate that has been very well studied [20]. In this study, infection of the skin models with *C. albicans* resulted in extensive hyphal growth associated with tissue dissociation and distinct cell damage after 48 h.

CAP treatment could suppress the infection depending on the treatment duration. So far, inactivation of *C. albicans* by plasma treatment has been studied mostly by culturing on agar or plastic surfaces [21,22,23]. Borges et al. showed a significant decline in hyphal growth after CAP treatment using a mouse model [24]. However, because of the morphological and immunological differences, these results can only be transferred to human skin to a limited extent [25]. To the best of our knowledge, this is the first study to describe *Candida* infection and CAP treatment in 3D-human skin models. The reduced hyphal activity could presumably be mediated by the free radicals contained in the plasma. The observed emission characteristics of the plasma source were slightly different from other dielectric barrier discharges studied in the medical field so far. Tučeková et al. investigated a diffuse coplanar surface barrier discharge (DCSBD) with an active plasma area of 200 mm × 80 mm and a discharge power of 400 W to inactivate *Escherichia coli* on PTFE surfaces [26]. These spectra are similarly dominated by the second positive system of molecular nitrogen N_2_ as observed here, but emissions of the first positive system also occurred. Furthermore, NO, OH, and N_2_^+^ were detected, dependent on the presence of H_2_O in the synthetic air. Another DBD in air employed for skin treatment showed primarily the second positive system of N_2_ and next to it the first negative system of N_2_^+^ [27]. In our investigations, OH emissions were not detectable in the plasma plume. However, it is possible that reactive oxygen and nitrogen species (ROS and RNS) formed by the interaction of the plasma with the treated material. It has been reported that excited N_2_ generation from CAP can increase reactive nitrogen species levels within cell growth media by producing nitrite ions (NO_2_^−^) from the proteins present [28]. In a liquid medium CAP can further generate H_2_O_2_, which has been found crucial for the antimicrobial effect [29]. In addition, it is likely that meaningful amounts of ozone and nitrogen oxides were generated at the interspace between the CAP generating LTCC plasma chip and the sample, as well as within the sample. It was found that ozone is effective in killing *C. albicans* [30], although much longer treatment periods were reported compared to our findings. Nitric oxide also exhibited broad spectrum antifungal activity in vitro after extended application times [31]. This suggests that the combination of reactive species in CAP can lead to oxidative stress, morphological changes, and cell membrane changes that more efficiently induce necrosis or apoptosis [32] in the yeast species. Plasma treatment has further been found to reduce the adherence of *Candida* as well as the transition from yeast to hyphae [33]. In addition, plasma is known to inhibit yeast growth by interfering with ergosterol biosynthesis [22].

The presence of pathogens is recognized by special “pattern recognition receptors” (PRRs). The binding of cell components, such as DNA, cell wall constituents, or RNA, activates the receptors [34,35]. Among others, epithelial cells respond to *C. albicans* colonization via the TLR-4-dependent signaling pathway that leads to the activation of the transcription factor NFκB. This results in the transcription of pro-inflammatory cytokines [36]. IL-1α is one of the first cytokines produced and secreted by keratinocytes in response to an infection. Large amounts of IL-1α are stored in the stratum corneum keratinocytes to initiate a rapid inflammatory and immune response upon infection [37]. Binding to a specific receptor causes activation of the transcription factor NFκB, which induces the production of other pro-inflammatory cytokines such as IL-6, IL-8, and TNFα [38]. It was demonstrated that infection of the skin models with *C. albicans* led to a significantly increased gene expression and secretion of IL-1α upon fungal recognition. Subsequently, other pro-inflammatory cytokine genes were also induced, such as *IL-6* and *CXCL8*, *TNFA, IL23*, and *GM-CSF*, with distinct functions in the inflammatory response following infection. IL-6 is a mediator of inflammation and activates the acute phase response leading to neutrophil activation [39], whereby IL-8 is responsible for the recruitment of neutrophils to the site of infection [40]. Moreover, TNFα stimulates the expression of chemokines and is involved in the recruitment of immune cells [41]. IL-23 is a pro-inflammatory cytokine that controls the expression of other cytokines and indirectly influences the production of antimicrobial peptides [42,43,44], and GM-CSF mediates the communication between immune cells and activates phagocytes [45]. After CAP treatment of infected skin models, a reduced expression and secretion of pro-inflammatory cytokines was observed. The decrease in cytokine expression and secretion was accompanied by the histologically observed decline in hyphal growth. To the best of our knowledge, this is the first report on CAP treatment of infected tissue or skin models with regard to pro-inflammatory cytokine modulation. So far, only Kitsin et al. have reported a decrease in *C. albicans* growth and reduced secretion of pro-inflammatory cytokines after treatment with an antifungal drug using a cutaneous candidiasis model [46].

Antimicrobial peptides (AMPs) represent the first line of defense in the humoral immune response to invasive microorganisms. AMPs are effector molecules that exert a direct antimicrobial function by interacting with microbial membranes or intracellular targets to disrupt the growth or reduce the viability of pathogens. AMPs are expressed by epithelial cells either constitutively or upon contact with the pathogen. AMP initiation is mediated by “pattern recognition receptors” (PRRs) such as the “toll-like receptors” (TLRs). Epithelial cells have evolved mechanisms to distinguish commensal *C. albicans* from invasive growth variants. The hyphal growth triggers the production and secretion of pro-inflammatory cytokines such as IL-6, IL-8, and TNFα. These cytokines are also relevant for the production of various AMPs [47]. In this study, the invasive growth of *C. albicans* resulted in the expression and secretion of pro-inflammatory cytokines associated with the induction of AMPs. Human beta defensin-2 (hBD-2) is produced by keratinocytes and is present in healthy skin. hBD-2 gene expression is induced by both IL-1α and TNFα. The antimicrobial effect is mediated by electrostatic interaction with the negatively charged membrane of the microorganisms [48]. Infection of the skin models resulted in an early increase in hBD-2 gene expression. This reaction was triggered by hyphal growth and the cellular pro-inflammatory response. There are few reports on the expression of hBD-2 in the context of *Candida* skin infection. Using a full-thickness human amniochorionic membrane, Zaga-Clavellina et al. were able to demonstrate secretion of hBD-2 after *Candida* infection [49]. Chadebech et al. observed increased expression of hBD-2 in a model of human reconstructed epidermis in response to LPS (lipopolysaccharide), a component of the cell wall of Gram-negative bacteria [50]. S100A7, also called psoriasin, is also constitutively expressed in the skin and increased during inflammation or infection. In addition to inducing pro-inflammatory cytokines and chemotactic properties, it acts as a zinc binder in *C. albicans* and induces cell death. Moreover, it impedes *C. albicans* binding by interacting with β-glucan [51]. In this study, *Candida* infection also led to the induction of S100A7 gene expression, and it is the first to show that CAP treatment of the infected skin models resulted in reduced S100A7 transcript levels, which were associated with reduction in hyphal growth. Another antimicrobial protein is RNase7, the dominant RNase in human skin, which is constitutively expressed but can also be induced by pro-inflammatory cytokines or microorganisms. In *C. albicans* it causes RNA cleavage and cell lysis [52,53]. Similar to the other AMPs, infection of the skin models in this study resulted in a significant increase in *RNASE7* gene expression. CAP treatment reduced the expression of *RNASE7*, which is the first time that this could be demonstrated for this antimicrobial peptide in connection with plasma treatment of infected cells or skin models.

In conclusion, this study confirmed the effectiveness of the tested CAP device against *C. albicans* infection in a 3D skin model for cutaneous candidiasis. So far, there are only few clinical studies on CAP treatment of yeast infections. Lipner et al. investigated the effect of plasma on onychomycosis in a pilot study, where, in addition to *T. rubrum*, the nails of some patients were colonized with *C. albicans*. They demonstrated the clinical efficacy and safety of the plasma treatment [54]. Since plasma has a high therapeutic potential, future studies should be conducted to examine the efficacy of plasma in candidiasis in more detail. Here, it was shown that infection with *C. albicans* causes expression and secretion of pro-inflammatory cytokines as well as the expression of antimicrobial peptides in a 3D human full skin model. CAP treatment of infected skin models resulted in a treatment duration-dependent reduction of hyphal growth associated with a decreased expression of the different infection markers. Such a model of cutaneous candidiasis provides an application-oriented approach for the testing of plasma devices. Moreover, the results demonstrated that the tested CAP device offers a suitable alternative treatment option for cutaneous *Candida* infections, providing a basis for future clinical investigations. CAP therapy could be a viable option for treatment of candidiasis, with a reduced risk for development of antimycotic resistance.

## 4. Materials and Methods

### 4.1. Plasma Device

The miniaturized ceramic atmospheric plasma source was manufactured using LTCC technology (low-temperature cofired ceramic). This enabled the production of very fine electrodes (150 μm) over the entire surface and their covering with a dielectric layer [9]. The high-frequency voltage was contacted via electrical vias in the back of the ceramic chip. The plasma was generated by barrier discharge and used ambient air as the working gas. This system had a very low power consumption of approx 5 W. The maximum temperature at the tip was below 40 °C.

### 4.2. Optical Emission Spectroscopy (OES)

The exited species of the plasma discharge were measured by optical emission spectroscopy in a wavelength range between 200 and 1100 nm. For the investigations a commercial EMICON MC spectrometer (Plasus GmbH, Mering, Germany) was used, with a spectral resolution (FWHM) of approx 1.5 nm. The distance between the sensor head and the ceramic of the plasma device was about 5 mm. Inside the senor head a collimator was installed to collect primarily the emissions perpendicular from the plasma source. The aperture diameter of the sensor head was 10 mm, so the measured spectra provided information averaged over the whole plasma area. The integration time was set to 1 s and the spectra were averaged over two measurements. In order to determine the emission lines the software SPECLINE (Plasus GmbH, Mering, Germany) was used.

### 4.3. Infection and Plasma Treatment of 3D-Skin Models

Three-dimensional skin models were manufactured as previously reported [16,17]. *C. albicans* (ATCC MYA-2876) was cultured on Sabouraud Dextrose Agar (SDA) plates (bioMérieux, Craponne, France). Yeast colonies were suspended in Sabouraud Glucose-Bouillon (Merck, Darmstadt, Germany) and cultured over night at 37 °C under vigorous shaking. The yeast suspension was washed twice in 0.9% NaCl, and the number of cells in the solution was determined by serial dilution followed by plating on SDA plates, which were incubated for 24 h at 37 °C. Colonies were counted and the microbial count (in colony-forming units/mL) of the yeast suspension was calculated. The skin models were infected with 10 μL of the *C. albicans* suspension (1 × 10^3^ cfu/mL). After 1 h incubation, infected skin models were treated with CAP for up to 40 s with a working distance of 1 mm. Sampling took place at 1, 24, and 48 h after treatment. For cytokine determination, supernatants were collected and stored at −20 °C. Skin models were transferred to a 4% formalin solution (Dr. K. Hollborn & Söhne, Leipzig, Germany) for histological examination. Skin models were further frozen in liquid nitrogen and stored at −80 °C for gene expression analysis.

### 4.4. Determination of Cytotoxicity

Cytotoxic effects were determined by measuring lactate dehydrogenase (LDH) in the supernatant of the skin models using a cytotoxicity detection kit (Roche, Basel, Switzerland). The assay was performed according to the manufacturer’s recommendations. The optical density at 490 nm was measured using a plate photometer (SPECTROstar Omega, BMG Labtech GmbH, Ortenberg, Germany). The fold LDH release was calculated relative to the control.

### 4.5. Determination of Cytokine Secretion

Cytokine release was quantified using human interleukin (IL)-6 (Mabtech, Stockholm, Sweden), IL-8, and IL-1α (R&D Systems, Minneapolis, MN, USA) enzyme-linked immunosorbent assay kits according to the manufacturers’ instructions. The optical density was measured at 450 nm with reference measurement at 620 nm using a plate photometer (SPECTROstar Omega, BMG Labtech GmbH, Ortenberg, Germany). Interleukin concentrations were calculated according to a standard curve using a 4-parameter fit with lin-log coordinates for optical density. The concentration was expressed as fold secretion compared to the control.

### 4.6. Histological Analysis

Formalin fixed models were dehydrated and embedded in paraffin blocks (Merck, Branchburg, NJ, USA) using standard histological protocols. Sections of 4 μm thickness were cut and mounted on slides. Before staining, the paraffin contained in the tissue section was removed by descending alcohol series. Dewaxing and staining with hematoxylin and eosin or periodic acid–Schiff (PAS)-staining were performed using the Leica Autostainer XL (Leica, Wetzlar, Germany) according to the manufacturer’s recommendations. The microscopic assessment was carried out using the Axio Scope A.1 microscope (Carl Zeiss, Jena, Germany). For documentation, photographs were taken with the digital camera AxioCam MRc (Carl Zeiss, Jena, Germany).

### 4.7. Quantification of Hyphal Growth

For this purpose, PAS staining was performed without counterstaining with hematoxylin. The microscopic assessment was carried out using the Axio Scope A.1 microscope (Carl Zeiss, Jena, Germany). For documentation, photographs were taken with the digital camera AxioCam MRc (Carl Zeiss, Jena, Germany). The area of the hyphal growth was determined in comparison to the unaffected area of the skin model using ImageJ.

### 4.8. Gene Expression Analysis

RNA was isolated using the RNeasy Mini Purification Kit (Qiagen, Hilden, Germany). cDNA was generated using 20 ng of isolated RNA and the High Capacity cDNA Reverse Transcription Kit (Thermo Fisher, Waltham, MA, USA). Real-time PCR was performed with the QuantiNova SYBR Green PCR Kit (Qiagen, Hilden, Germany). PCR products were amplified using the following steps: initialization at 95 °C for 180 s, followed by 40 cycles of 95 °C for 5 s, 57 °C for 10 s, and 72 °C for 10 s. The fold-change in specific gene expression was calculated based on the 2^−ΔΔCT^ method with β-actin as the housekeeping gene. Primer sequences are listed in Table 1.

### 4.9. Statistics

Experiments were performed in duplicate, and each sample was measured in two replicates. Data evaluation was carried out with Excel 2010 (Microsoft Corp., Redmond, WA, USA) and OriginLab 9.0 (OriginLab Corp., Northampton, MA, USA). Statistical analyses were performed using SPSS Version 27 (IBM Corp., Armonk, NY, USA). Statistical significance was determined using the non-parametric Mann–Whitney U test. Significant (* *p* < 0.05), very significant (** *p* < 0.01), and highly significant (*** *p* < 0.001) deviations are displayed.

## Figures and Tables

**Table 1:** Primer sequences used for real-time polymerase chain reaction.

Gene Name	Primer Sequence (5’-> 3’)	
	Forward Primer	Reverse Primer
Interleukin-6/*IL6*	CCA CCG GGA ACG AAA GAG AA	GAG AAG GCA ACT GGA CCG AA
Interleukin-8/*CXCL8*	QT00000322 (Qiagen)	
Interleukin-1α/*IL1A*	CGC CAA TGA CTC AGA GGA AGA	AGG GCG TCA TTC AGG ATG AA
Interleukin-23/*IL23*	QT00204078 (Qiagen)	
TNFα/*TNFA*	QT00029162 (Qiagen)	
GM-CSF/*GM-CSF*	TGA ACC TGA GTA GAG ACA CTG C	GCT CCT GGA GGT CAA ACA TTT C
hBD2/*HBD2*	CCA GCC ATC AGC CAT GAG GGT	GGA GCC CTT TCT GAA TCC GCA
S100A7	GTC CAA ACA CAC ACA TCT CAC T	TCA TCA TCG TCA GCA GGC TT
RNase7/*RNASE7*	QT00239463 (Qiagen)	
β-Actin	QT01680476 (Qiagen)	

## Data Availability

All data generated or analyzed during this study are included in this article. Further inquiries can be directed to the corresponding author.

## Supplementary Materials

The following supporting information can be downloaded at: https://www.mdpi.com/article/10.3390/antibiotics12050933/s1, Figure S1: Histological analysis of hyphal growth: 3D skin models were infected with C. albicans and treated with CAP for 5, 10 and 40 s. *C. albicans* hyphal growth was visualized after 1, 24 and 48 hours using PAS staining.
