# Supplementary material for: Cold Atmospheric Plasma Exerts Antimicrobial Effects in a 3D Skin Model of Cutaneous Candidiasis
Source: Antibiotics (Basel). 2023 May 19;12(5):933. doi: 10.3390/antibiotics12050933 (PMC10215140; doi:10.3390/antibiotics12050933)
Supplement: Supplementary file 1 [file antibiotics-12-00933-s001.zip › antibiotics-2340416-supplementary.pdf]

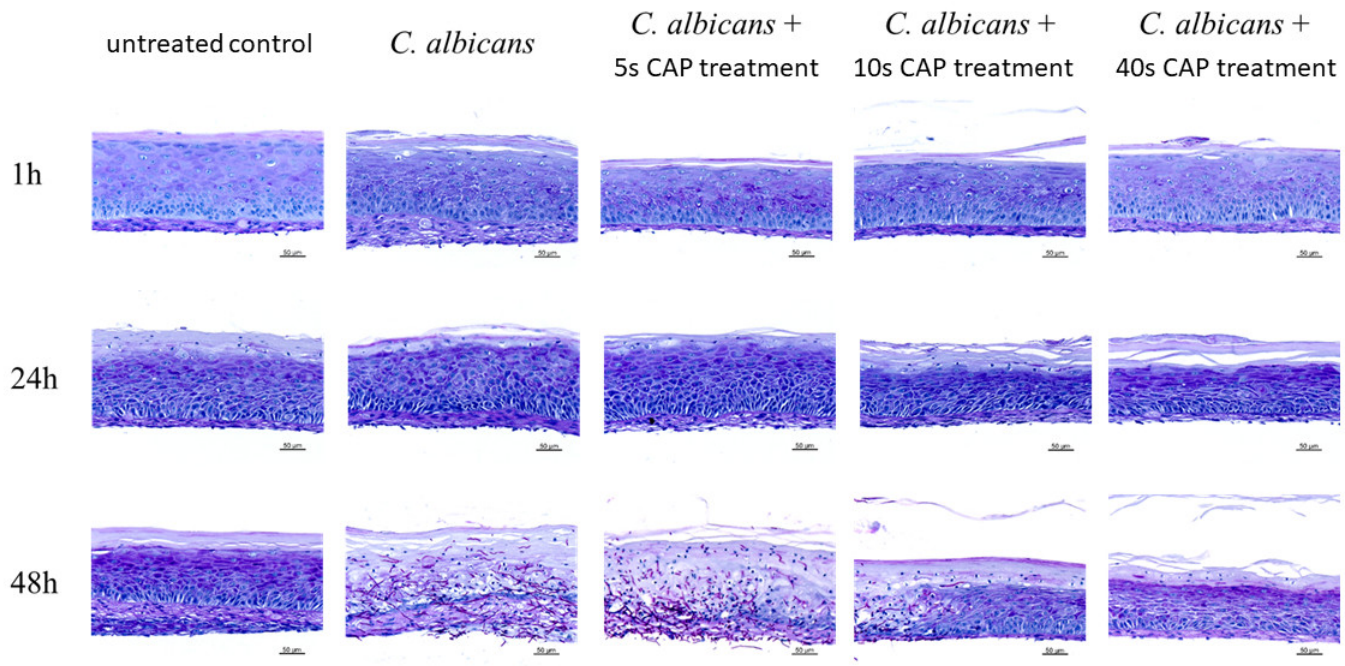

Figure S1. **Histological analysis of hyphal growth:** 3D skin models were infected with *C. albicans* and treated with CAP for 5, 10 and 40 s. *C. albicans* hyphal growth was visualized after 1, 24 and 48 hours using PAS staining.

Commented [M1]: We removed the title here from the figure, please check it.
